# F115. INSIGHT AND MANIC SYMPTOMS IN PATIENTS WITH CHRONIC SCHIZOPHRENIA IN THE KOREAN COMMUNITY

**DOI:** 10.1093/schbul/sby017.646

**Published:** 2018-04-01

**Authors:** Duk-In Jon, Bo-Hyun Yoon, Sang-Yeol Lee, Kwanghun Lee, Won-Myong Bahk, Beomwoo Nam, Sung-Yong Park, Min-Kyu Song

**Affiliations:** 1 Hallym University Sacred Heart Hospital; 2 Naju National Hospital; 3 Wonkwnag University School of Medicine and Hospital; 4 Dongguk University Hospital; 5 Yeouido St. Mary’s Hospital, The Catholic University of Korea; 6 Konkuk University; 7 Keyo Hospital; 8 Yesan Mental Health Clinic

## Abstract

**Background:**

Many studies have highlighted the similarity of the symptoms between bipolar disorders and schizophrenia. Moreover, there are no pathognomonic symptoms that can differentiate these two disorders, and 9% of schizophrenia patients have experienced a manic syndrome in their lifetime. Insight about their symptoms and illness is very important factor for the differential diagnosis and the management in schizophrenia. To examine the relationship among the insight, the psychotic and manic symptoms, and clinical variables in patients with chronic schizophrenia.

**Methods:**

Seventy-four participants (male 44, female 30) with chronic schizophrenia in community mental health facilities have been evaluated with the Scale to assess Unawareness of Mental Disorder (SUMD), the Mood Disorder Questionnaire (MDQ), and the Brief Psychiatric Rating Scale (BPRS).

**Results:**

The mean number of previous admission was 3.85. Their drug adherence was favorable (6.73 day/week). Mean CGI-S score was 3.8. Thirty-five percent of subject were MDQ positive (cutoff point = 7 or more). Among SUMD, “awareness of effect of medication” showed significant negative correlation (r = -0.33) with total MDQ score not with total BPRS score. The negative correlation was more obvious in participants with negative MDQ (total MDQ score 6 or less, r = -0.31). Several MDQ items (irritability, r = -0.25; decreased sleep, r = -0.27; thought racing, r = -0.28; and easy distractibility, r = -0.40) negatively correlated with “awareness of effect of medication”. In contrast, only one item (guilt feeling, r = -0.27) of BPRS revealed this correlation. Individual items in MDQ and BPRS rarely correlated with each other. Total MDQ score was not correlated with duration of illness and medication adherence.

**Discussion:**

Manic symptoms were frequently detected even in schizophrenia as reported in previous studies. This made it difficult to differentially diagnose the disorder using only the total MDQ score. There was possible relationship between these manic symptoms and their insight. Identifying manic symptoms in schizophrenia would be considerable in clinical setting.

